# 2012 Update in addiction medicine for the generalist

**DOI:** 10.1186/1940-0640-8-6

**Published:** 2013-03-13

**Authors:** Darius A Rastegar, Hillary V Kunins, Jeanette M Tetrault, Alexander Y Walley, Adam J Gordon

**Affiliations:** 1 Johns Hopkins School of Medicine, Baltimore, MD, USA; 2 Montefiore Medical Center/Albert Einstein College of Medicine, Queens, NY, USA; 3 Yale University School of Medicine, New Haven, CT, USA; 4 Boston University School of Medicine, Boston, MA, USA; 5 University of Pittsburgh and VA Pittsburgh Healthcare System, Pittsburgh, PA, USA

**Keywords:** Primary care, Alcoholism, Addictive behavior, Drug abuse, Substance-related disorders, Screening and brief intervention

## Abstract

This article presents an update on addiction-related medical literature for the calendar years 2010 and 2011, focusing on studies that have implications for generalist practice. We present articles pertaining to medical comorbidities and complications, prescription drug misuse among patients with chronic pain, screening and brief interventions (SBIs), and pharmacotherapy for addiction.

## Introduction

The goal of this paper is to identify and examine important recent advances in addiction medicine in the medical literature that have implications for the care of patients in primary care or other generalist settings. To accomplish this, the authors selected articles in the field of addiction medicine and summarized, critically appraised, and examined the articles in context of their implications for generalist practice, employing methodology used in prior updates [1-3]. During an initial review, we identified articles through an electronic Medline search (limited to human studies and in English) using search terms for alcohol and other drugs of abuse from January 2010 to January 2012. From the citations, the authors selected articles for more intensive review. After this initial review, we searched for other literature in web-based or journal resources (e.g., Alcohol, Other Drugs, and Health: Current Evidence [http://www.aodhealth.org], ACP Journal Club, and tables of contents of relevant journals). Through a consensus process, the authors agreed collectively on the important articles regarding addiction medicine that have implications for practice for generalist clinicians.

We have divided our review into four broad categories: medical comorbidities and complications, prescription drug misuse among patients with chronic pain, screening and brief interventions (SBIs), and pharmacotherapy for addiction.

### Medical comorbidities and complications

Medical complications are common for patients who consume unhealthy amounts of alcohol, those with alcohol dependence, and those who use illicit drugs [4-6]. In the United States, unhealthy alcohol consumption (i.e., consuming quantities that place a person at risk for medical harm) is defined as >14 standard drinks (14 g alcohol) weekly and >4 drinks on one occasion in the last year for men, and >7 standard drinks weekly and >3 drinks on one occasion in the last year for women and those over 65 years of age [7]. Consumption of this amount can impart significant harm, including cardiovascular, gastroenterological, and neurological morbidity. Heavy episodic (“binge”) consumption may impart specific harms. Furthermore, when alcohol abuse and alcohol dependence occur, additional specific complications can arise, including delirium tremens. Identifying and intervening on this condition early may reduce subsequent complications. Medical complications from illicit substance use can also include cardiovascular morbidity.

### Are alcohol screening scores on the AUDIT-C associated with surgical complications?

Bradley KA and colleagues [8].

This retrospective cohort study examined surgical outcomes among male patients from the Veterans Affairs (VA) health-care system who had been screened for unhealthy alcohol use using the three Alcohol Use Disorders Identification Test - Consumption (AUDIT-C) questions. The outcome was any surgical complication within 30 days post-operatively. The rate of post-operative complications ranged from 5.6% (95% confidence interval [CI], 4.8-6.6%) for patients with AUDIT-C scores between 1 and 4, to 14% (95% CI, 8.9-21.3%) among patients with AUDIT-C scores of 11 or 12. The main implication of this study was that an elevated pre-operative AUDIT-C score can help identify patients who are at risk for postoperative complications. This finding suggests that these patients should be aggressively counseled about cutting back on alcohol use prior to elective surgery; however, because of the observational study design, an intervention study is needed to confirm this recommendation.

### Is consistent drinking associated with risk of atrial fibrillation?

Kodama S and colleagues [9].

Although episodic alcohol consumption increases risk for atrial fibrillation, it is not known whether consistent alcohol consumption does. In this meta-analysis of 14 cohort and case–control studies, the pooled odds ratio (OR) for any alcohol use and development of atrial fibrillation was 1.5 (95% CI, 1.31-1.74). Atrial fibrillation relative risk was 1.08 for each 10 g per day of alcohol consumed (95% CI, 1.05-1.10). Based on this meta-analysis, regular drinking was associated with atrial fibrillation in a dose–response fashion, even at alcohol levels below current definitions of risky use—a new and important finding.

### Do pattern and type of alcohol use affect risk for myocardial infarction and coronary death?

Ruidavets JB and colleagues [10].

Previous research has found an inverse relationship between alcohol consumption and coronary heart disease. This prospective study followed men in France and Ireland (aged 50–59 years) for 10 years. In examining the association between pattern and type of alcohol consumption, the authors found that binge versus non-binge drinkers had a hazard ratio (HR) for myocardial infarction (MI) or coronary death of 1.97 (95% CI, 1.21-3.22). Wine drinkers, as compared with drinkers of other alcohol types, had an HR of 0.57 (95% CI, 0.38-0.85) for MI or coronary death. The finding of an association between binge drinking and cardiovascular health suggests that both pattern of drinking and overall consumption contribute to cardiovascular risk. That types of alcohol (beer and liquor) may adversely affect coronary health is suggestive, but type of alcohol may serve as a marker for other lifestyle factors for which this observational study could not fully control.

### Are there identifiable risk factors for seizures and delirium tremens among patients hospitalized for alcohol withdrawal?

Eyer F and colleagues [11].

In this retrospective study of German adults hospitalized for alcohol detoxification, all patients were treated with withdrawal-score–guided benzodiazepines and antiepileptics. Of 827 adults, 5.6% developed delirium tremens (DTs), and 7.4% had seizures. Risk of DTs increased with a past structural brain lesion (OR, 5.8; 95% CI, 2.6-12.9) and decreased with higher platelet count (OR, 0.42 per 100,000 platelets; 95% CI, 0.26-0.69) and serum potassium (OR, 0.33 per 1 mmol/L; 95% CI, 0.17-0.65). Risk factors for seizures also included having a past structural brain lesion (OR, 6.0; 95% CI, 3.0-14.1). Other risks for seizure during admission included delayed peak withdrawal severity (OR, 1.2 per 10 hours; 95% CI, 3.0-14.1), and seizure as a cause of admission (OR, 2.6; 95% CI, 1.4-4.8). Only low serum potassium was identified as a modifiable risk factor in this study, although other risk factors, such as prior head trauma or low platelets, could alert clinicians to monitor these patients more carefully.

### Are beta-blockers safe in patients with chest pain and recent cocaine use?

Rangel C and colleagues [12].

Prior physiologic studies had suggested that the use of beta-blockers concurrently with cocaine may lead to coronary vasoconstriction [13]; however, the clinical significance of this observation has not been established. Nevertheless, American Heart Association guidelines have recommended against the use of beta-blockers in patients with chest pain who have recently used cocaine [14].This retrospective cohort study examined mortality among patients who presented to the emergency department with chest pain and had a urine drug test positive for cocaine [12]. Although the admissions were consecutive, urine drug tests were not performed in a systematic fashion. Despite this limitation, the study’s findings are important: there was no detected association between receipt of a beta-blocker and mortality. Patients who received a beta-blocker in the emergency department had an HR of 0.97 (95% CI, 0.53-1.79) for mortality, and patients who were discharged on a beta-blocker had an HR of 0.90 (95% CI, 0.47-1.71) for all-cause mortality as compared with those not given beta-blockers. In a secondary analysis, discharge on a beta-blocker was associated with a 70% reduction in cardiovascular death (HR, 0.29; 95% CI, 0.09-0.98). This study finds no evidence of increased harm among patients with chest pain and recent cocaine use treated with beta-blockers, and lower risk of cardiovascular death among cocaine users discharged on beta-blockers. However, patients were not systematically tested for cocaine; untested cocaine users in the sample may have elevated the risk of adverse outcomes within the comparison group, thereby biasing the results towards the null. The optimal role of beta-blockers among patients with chest pain and cocaine use remains unclear and requires further study.

### Implications for practice

Medical harm does occur with unhealthy alcohol and illicit drug use. The relationship between alcohol use and cardiovascular health remain an area for ongoing investigation; furthermore, best management practices for cocaine-related chest pain should continue to be investigated. Regardless, for clinicians, the association of harms from unhealthy alcohol use and illicit drug use may encourage clinicians to screen and offer tailored advice to patients at risk from these medical complications. In particular, since alcohol is a leading cause of preventable death and disease in the United States, clinicians should counsel all patients that excessive consumption of alcohol may impart significant medical and social complications.

### Prescription drug misuse among patients with chronic pain

Broader prescribing of opioids for chronic pain has come with substantial increases in opioid-related overdose fatalities and hospitalizations. Prescribers need better tools to identify patients who are at risk for opioid misuse and overdose, to monitor patients receiving opioid pain medication, and to treat pain safely and effectively in patients who have both chronic pain and substance use disorders (SUDs).

### What characteristics are associated with prescription drug use disorders among patients treated for chronic pain?

Liebschutz JM and colleagues [15].

Liebschutz and colleagues surveyed 597 urban primary-care clinic patients with chronic pain taking any analgesic to identify risk factors for a lifetime prescription drug use disorder (PDUD) as defined using *Diagnostic and Statistical Manual of Mental Disorders*, 4th ed. (DSM IV) criteria. The prevalence of PDUD was 18% (110 of 597 patients surveyed). Risk factors for PDUD were as follows: time in jail (adjusted OR, 5.1; 95% CI, 2.8-9.3), greater pain-related limitations (adjusted OR, 3.8; 95% CI, 1.2-11.7), current smoking (adjusted OR, 3.6; 95% CI, 2.0-6.2), family history of substance abuse (adjusted OR, 3.4; 95% CI, 1.9-6.0), white race (adjusted OR 3.2; 95% CI, 1.7-6.0), male gender (adjusted OR, 1.9; 95% CI, 1.1-3.5), and post-traumatic stress disorder (adjusted OR, 1.9; 95% CI, 1.1-3.4). All patients with lifetime PDUD had at least two of these risk factors.

### How best to monitor pain patients for opioid misuse?

Starrels JL and colleagues [16].

Paulozzi LJ and colleagues [17].

Tools recommended for prescribers to monitor patients for prescription opioid misuse include treatment agreements, urine drug testing [18,19], and utilization of prescription drug monitoring programs [20]. Starrels and colleagues [16] conducted a systematic review of the effectiveness of opioid treatment agreements (OTAs) and urine drug testing (UDT) to reduce opioid misuse among outpatients prescribed opioids for noncancer pain. Of the 11 studies that met inclusion criteria, three evaluated OTAs only, one evaluated UDT only, and seven evaluated both. All studies were observational and were rated as poor-to-fair in quality. The four studies where there was a comparison group reported a range of 7-23% absolute-risk reduction in opioid misuse. Several studies included multicomponent interventions that were not representative of common clinical practice. Poor study quality and variation in practice setting and interventions did not permit performing a meta-analysis.

Paulozzi and colleagues [17] conducted a national interrupted time series analysis of state prescription drug monitoring programs (PDMPs) to determine associations between PDMPs, opioid overdose death rates, and prescription opioid distribution rates. Between 1999 and 2005, both opioid overdose death rates and prescription opioid distribution rates tripled. Results of the analysis indicated that PDMPs were not a significant predictor of opioid overdose mortality (p = 0.3) or opioid distribution (p = 0.6). States with PDMPs had higher Schedule-III opioid distribution, whereas states without PDMPs had higher Schedule-II opioid distribution. In comparison with other PDMP and non-PDMP states, three large states with PDMPs and tamper-resistant prescription forms (California, New York, and Texas) did have a lower combined opioid overdose death rate (1.65 deaths per 100, 000 person years versus 3.13 for other PDMP states and 2.20 for non-PDMP states) and lower opioid distribution rates (251 morphine milligram equivalents per person per year versus 362 for other PDMP states and 342 for non-PDMP states).

### How should patients with chronic pain and a history of substance use be treated?

Morasco BJ and colleagues [21].

A primary-care–based chronic disease management program for chronic pain, including psychological assessment, telephone support, a four-session workshop with a psychologist, and physical therapy, can improve function, pain, and mood [22]. Morasco and colleagues [21] conducted a secondary analysis of a randomized controlled trial to determine whether patients with an SUD history benefited similarly from the chronic pain disease management program. The 20% of the study sample (72 of 362 patients) with an SUD history were younger, less likely to be married, less physically functional, more likely to be treated with opioids, and more likely to have depression or post-traumatic stress disorder. At 12 months, patients with an SUD history assigned to the intervention group had similar pain improvement to intervention patients with no SUD history (adjusted OR, 1.06; 95% CI, 0.37-3.01), whereas there was less pain improvement among patients with an SUD history assigned to the control group compared with non-SUD control-group patients (adjusted OR, 0.30; 95% CI, 0.11-0.82).

### Implications for practice

Awareness of key characteristics, including previous incarceration, more severe pain-related limitations, smoking, family history of substance abuse, white race, male gender, and post-traumatic stress disorder should help prescribers identify patients who are at higher risk for PDUD. Opioid treatment agreements (OTAs) and urine drug testing (UDT) are guideline-recommended tools to reduce prescription opioid misuse, but the evidence for their effectiveness is not strong, and understanding how best to incorporate them into busy primary care practices requires more research. Prescription drug monitoring programs (PDMPs) are another tool increasingly available, but have yet to show an overall impact on overdose rates. They may shift prescribing towards medications that are not as closely monitored. When combined with other prescription monitoring efforts, PDMPs may have a positive impact on opioid overdose and prescribing rates. For patients with chronic pain and known SUD histories, more intensive and comprehensive chronic pain management programs should be offered to improve pain control, because with treatment as usual, patients with a history of SUD have worse pain control than those without.

### Screening and brief interventions

The clinical procedures of screening for unhealthy alcohol and substance use, providing brief interventions to reduce unhealthy substance use, and referring for treatment patients in need of specialty treatment are evidence-based practices that are being promoted by research, policy, and service organizations in the United States [23]. This cluster of clinical activities (screening, brief intervention, and referral to treatment [SBIRT]) has been advocated to encourage screening and improve treatment and care coordination between general and specialty addiction services [24,25]. An emerging body of literature aims to facilitate SBIRT implementation [26,27]. Recent literature has examined the ability to screen for drug use and examine the quality of screening of unhealthy alcohol consumption in primary care settings. Once identified through the screening process, recent work has examined the efficacy of brief interventions for primary care patients with heavy drinking and alcohol dependence and assessed the capability of nonphysicians to perform brief interventions.

### How does a busy clinician screen for drug use?

Smith PC and colleagues [28].

Drug use, including illicit drug use and nonmedical use of prescription medications, is a common but potentially under-recognized problem among primary care patients. Smith and colleagues sought to validate a single question screening test for drug use and drug use disorders in primary care. They asked a sample of primary care patients, “How many times in the past year have you used an illegal drug or used a prescription medication for nonmedical reasons?” A positive response was any affirmative answer, and this response was compared with the presence of past 12-month documented drug use or drug use disorder (abuse or dependence) per DSM IV criteria [29]. The Drug Abuse Screening Test [30] was also administered, and drug use was confirmed through oral testing for common illicit substances. Using these measures, the single-item question was 100% sensitive (95% CI, 90.6%-100%) and 73.5% specific (95% CI, 67.7%-78.6%) for the detection of a current drug use disorder. The authors concluded that the single screening question accurately identified drug use in a sample of primary care patients, supporting its usefulness in primary care. As primary care providers increasingly are pressed to prevent, identify, and manage risky behaviors among their patients, the ability of a brief screening test for drug use and drug use disorders may encourage greater recognition—and subsequent treatment—among their patients.

### What is the quality of screening for unhealthy alcohol consumption in clinical practice?

Bradley KA and colleagues [31].

Alcohol screening questionnaires have typically been examined and validated in environments where the patient completes the questionnaire or an investigator, through an interview process, administers it. Little is known about the performance of these instruments when implemented as part of routine clinical care. The environment in which screening is administered and the quality of administration may impact the performance of the instrument. Bradley and colleagues sought to compare the result of an alcohol screening questionnaire conducted as part of routine outpatient clinical care to results of the same alcohol screening questionnaire when completed in a mailed survey to identify factors associated with discordant screening results. They examined a national sample of 6861 VA primary care outpatients who completed the three Alcohol Use Disorder Identification Test - Consumption (AUDIT-C) questions [32,33] during a clinical visit and then, within 90 days, received, completed, and returned the AUDIT-C as part of a mailed survey. The investigators used multivariable logistic regression to estimate the prevalence of discordance in the different patient subgroups based on demographic, clinical, and primary care environment characteristics and temporal associations (i.e., the order of the administration of the in-clinic and mailed assessments). Overall, 390 of the clinical AUDIT-C screens and 765 of the survey AUDIT-C screens were positive (AUDIT-C score ≥5); 61% of the positive survey screens had negative clinical screening results. Discordance was significantly higher among African-American patients compared to Caucasian patients, among patients who had a positive survey screen, and those who received care in certain VA care networks. The authors concluded that use of validated alcohol screening questionnaires does not by itself ensure the quality of alcohol screening, while the results imply that screening for alcohol consumption using the AUDIT-C may be more effective when self-administered versus provider-administered. Perhaps improved quality of provider-administered screening will improve the quality of screening in practice, and might have reduced the discordant clinic and survey screening results found in this paper.

### Do brief interventions work, and, if so, for whom and by whom?

Saitz R [34].

As SBIRT is promoted in general medical environments, screening for unhealthy alcohol use is bound to identify patients with alcohol abuse and alcohol dependence. Unfortunately, while many brief intervention efficacy studies have shown an effect of reducing alcohol consumption among patients who drink at unhealthy levels, these same studies have generally excluded patients with alcohol abuse and dependence. In real-world settings, clinicians may identify patients with alcohol abuse and dependence, but are brief interventions efficacious for these patients? Saitz performed a systematic review of brief intervention trials in the literature to examine this question in primary care settings. Of the 16 randomized controlled studies identified that compared outcomes among adults with unhealthy alcohol use identified by screening who received a brief intervention in a primary care setting with those who received no intervention, 14 excluded some or all subjects with very heavy alcohol use or with alcohol abuse or dependence diagnoses. In the two remaining studies [35,36], brief interventions among subjects with alcohol dependence were not found to be effective at reducing alcohol severity scores or drinking. Saitz concludes that, while alcohol screening and brief intervention have efficacy in primary care for patients with unhealthy alcohol use, there is a lack of evidence for efficacy among those with abuse and dependence. This study highlights the need to develop new approaches to help patients with heavy alcohol use, abuse, or dependence identified in primary care practices via alcohol screening. If screening and brief interventions are to continue to be advocated widely in practice, interventions or referral mechanisms for patients with severe alcohol problems should be tested and implemented.

Sullivan LE and colleagues [37].

There are a variety of provider-, system-, and patient-level barriers to SBIRT delivery; although not exclusively, the majority of provider-level barriers identified are from the *physician* perspective. These include concerns about lack of time and training for performing SBIRT, perception or presence of more compelling clinical issues, underutilization or lack of awareness of effective screening and treatment strategies for alcohol and other drugs, and concerns about patient privacy and potential damage to the patient-provider relationship [38,39]. Additionally, providers may perceive that screening and intervention for unhealthy alcohol use is simply ineffective, unsatisfying, uncomfortable, or not within their role responsibilities [40]. As early as 2003, the US Institute of Medicine recognized that “all health professionals should be educated to deliver patient-centered care as members of an interdisciplinary team, emphasizing evidence-based practice, quality improvement approaches, and informatics” [41]. The implementation of SBIRT is an ideal modality to make multidisciplinary approaches to the provision of health care a reality [40]. Sullivan and colleagues [37] performed a systematic review of the English literature and identified 13 studies where nonphysician practitioners performed brief interventions. Seven of these studies, encompassing a total of 2633 subjects, were included in a meta-analysis. The authors found that patients who received nonphysician interventions consumed 1.7 fewer standard drinks per week than control subjects (95% CI, 0.03-3.5) and concluded that nonphysician brief interventions are modestly effective at reducing drinking in primary care settings. Although it is unclear whether nonphysician interventions are as effective as those performed by physicians, this study highlights the importance of nonphysician providers in the identification and treatment of patients with addiction in primary care. Models of care should incorporate training and flexibility for nonphysician providers to perform brief interventions for unhealthy alcohol use in practice.

### Implications for practice

Screening, brief intervention, and referral to treatment has been widely advocated to reduce the harms associated with unhealthy alcohol consumption. These studies indicate that screening for drug use may be possible, that the way we screen for unhealthy alcohol consumption may be important, that brief interventions for alcohol abuse or dependence may not be effective, and that brief interventions can be performed by nonphysicians. Clinicians must make careful choices regarding how to screen, what to screen for, and what interventions are appropriate for patients with alcohol and drug use. The evidence base to steer these choices has yet to be established for the gamut of alcohol and drug use disorders that generalist physicians encounter in clinical practice.

### Pharmacotherapy for addiction

#### Pharmacotherapy for opioid dependence

For many years, methadone, an opioid agonist, was the main pharmacologic treatment option for opioid dependence, but its availability in the United States was limited to licensed programs. The introduction of another opioid agonist in the form of sublingual buprenorphine, which can be prescribed in office-based practices, has greatly expanded access to treatment for individuals with opioid dependence. Although the effectiveness of buprenorphine has been demonstrated in clinical trials [42] and confirmed in observational studies [43], there are still questions about how best to deliver this treatment and its impact on the treatment of other conditions. The opioid antagonist naltrexone has been available as a treatment for opioid dependence for many years, but its use has been limited, particularly in countries where agonist treatment is available. The recent FDA approval of an injectable extended-release formulation of naltrexone offers a new pharmacologic application of this medication.

#### Does adjunctive counseling improve outcomes for outpatients receiving buprenorphine for opioid dependence?

Weiss RD and colleagues [44].

Although office-based buprenorphine has been shown to be an effective treatment for opioid dependence, the impact of adjunctive counseling remains uncertain [45]. Weiss and colleagues [44] investigated this question in a randomized controlled trial that recruited 653 prescription-opioid–dependent adults. All subjects received buprenorphine and medical management and were randomly assigned to receive either adjunctive counseling or no additional treatment. The main endpoint was “successful outcome” defined by a number of factors, including retention in treatment, self-reported opioid use, and the results of UDT. There were two stages to the trial: the first stage included four weeks of treatment with eight weeks of subsequent follow-up. Subjects who did not have successful outcomes after the first stage then entered a second stage including 16 additional weeks of treatment and eight additional weeks of follow-up. Over the course of both stages, there was no significant difference in outcomes between patients assigned to adjunctive counseling and those who were not. Only 6.6% of the subjects were successful in the first stage. During the second stage, 49% of subjects were successful while receiving buprenorphine treatment, but only 8.6% were able to maintain success in the eight weeks after they were tapered off of buprenorphine.

#### Can outpatient buprenorphine treatment be effectively delivered in a collaborative model of care?

Alford DP and colleagues [46].

To expand access to buprenorphine, there is a need for more physicians to provide this treatment. Barriers include the demand on time and lack of ancillary support [47,48]. A collaborative care model is one way to address these barriers. Alford and colleagues [46] developed a model in which physicians worked with nurses and other clinical staff to provide office-based buprenorphine. In this paper, they presented the one-year outcomes of 408 patients who were admitted to the program over a five-year period. Success was defined as remaining in treatment for 12 months or tapering off of treatment after six months of abstinence and treatment adherence. Overall, 51% of participants were considered to be successful; 49% remained in treatment, and 2% were successfully tapered. Another 6% transferred to methadone maintenance treatment, and the remaining 42% were considered unsuccessful either by dropping out of treatment (30%) or nonadherence despite more intensive treatment (increased visits and more intensive counseling) (12%). Older age, being employed, and prior illicit buprenorphine use were associated with treatment success.

#### Does integration of buprenorphine treatment with other medical care improve access to treatment and health outcomes?

Lucas GM and colleagues [49].

Another potential benefit of office-based buprenorphine is the engagement of patients in medical care and improvement in medical treatment outcomes. Lucas and colleagues recruited 93 subjects from 2005 to 2009 and randomly assigned them to buprenorphine treatment in an HIV clinic or referral to community-based addiction treatment. Subjects assigned to clinic-based buprenorphine were significantly more likely to initiate buprenorphine treatment within two weeks (84% versus 11%), to remain in treatment for 12 months (74% versus 41%), and had a higher median number of visits with their HIV provider (3.5 versus 3.0). They were also less likely to have an opioid-positive UDT (44% versus 65%) or cocaine-positive UDT (51% versus 66%). On the other hand, there were no significant differences in months of antiretroviral treatment, baseline CD4 cell count, HIV viral load, or proportion of patients with one or more emergency department visits or hospitalizations.

#### Is extended-release naltrexone an effective treatment for opioid dependence?

Krupitsky E and colleagues [50].

In 2010, the FDA approved extended-release injectable naltrexone for the treatment of opioid dependence. This approval was partly based on the results of a trial conducted at 13 addiction treatment centers in Russia, where opioid agonist treatment is prohibited. In this 24-week trial, Krupitsky and colleagues randomly assigned 250 opioid-dependent subjects who had completed inpatient detoxification to monthly injections of an extended-release naltrexone (XR-NTX) or placebo. Subjects assigned to XR-NTX were significantly more likely to complete the trial (53% versus 38%) and were more likely to be abstinent from opioids during weeks five through 24 (36% versus 23%). There was also a significantly greater reduction in opioid craving among those subjects receiving XR-NTX.

#### Implications for practice

The availability of buprenorphine for office-based treatment of opioid dependence has expanded its use. Weiss and colleagues’ study [44] confirms the effectiveness of this treatment but also shows that short-term treatment is generally not effective. Their finding suggests that adjunctive counseling offers little additional benefit, though it should be noted that their subjects were largely employed, well-educated, and had relatively brief histories of substance abuse; their findings may not apply to disadvantaged individuals or those with longer standing histories of addiction. Alford and colleagues [46] offer a model that may help expand treatment and include physicians who would probably not be able to provide office-based buprenorphine otherwise; in addition, this model is consistent with the team-based care approach incorporated into the medical home, an overall strategy to improve the quality and patient-centeredness in primary care. Lucas and colleagues [49] provide further evidence to support the effectiveness of office-based buprenorphine treatment and the integration of treatment with medical care. Although they failed to find significant improvements in clinical outcomes, other observational studies have found a correlation between initiation of buprenorphine and CD4 lymphocyte counts [51].

Naltrexone is another option for the treatment of opioid dependence, particularly for individuals in countries or professions that do not permit agonist treatment. However, its effectiveness is fairly modest and has never been compared with full or partial agonist treatment.

#### Pharmacotherapy for alcohol dependence

Currently, there are three medications approved by the US Food and Drug Administration for the treatment of alcohol dependence: disulfiram, an aldehyde dehydrogenase inhibitor approved in 1951; naltrexone, an opioid antagonist approved in oral form in 1995 and in depot form in 2006; and acamprosate, a GABA agonist/glutamate antagonist approved in 2004. Other agents under investigation for the treatment of alcohol dependence include two antiepileptics, topiramate and baclofen. Recent literature investigating pharmacotherapies for alcohol dependence have focused on opioid antagonists, acamprosate, topiramate, and baclofen.

#### Are opioid antagonists an effective treatment for alcohol dependence?

Rösner S and colleagues [52].

Prior reviews and meta-analyses suggest a small-to-moderate effect of opioid antagonists, mainly naltrexone, in preventing relapse to heavy drinking among people with alcohol dependence. A prior systematic review [53], published in 2005, included data from 29 studies and found that short-term treatment with naltrexone decreased relapse to alcohol use in 36% of subjects (number needed to treat [NNT] = 7) and decreased return to heavy drinking in 13% (NNT = 12). These data were limited by short-term treatment duration, small sample sizes, and lack of data on psychosocial benefits of treatment. Rösner and colleagues [52] updated this review, pooling data from 50 published randomized controlled trials of opioid agonists that included 7793 subjects. Primary outcomes were return to any drinking, return to heavy drinking, and number of drinking days. Most trials (N = 43) included data on oral naltrexone, four focused on injectable extended-release naltrexone, and three focused on nalmefene. Follow-up ranged from four to 52 weeks. Oral naltrexone, compared with placebo, decreased return to any drinking (71% versus 74%; relative risk [RR], 0.96; 95% CI, 0.92-1.00), decreased return to heavy drinking (51% versus 61%; RR 0.83; 95% CI, 0.76-0.90), and reduced drinking days by four days per month (95% CI, 2.04-5.75). Side effects were more common with naltrexone than with placebo. Depot naltrexone and nalmefene had similar efficacy to oral naltrexone.

Lee JD and colleagues [54].

This 12-week, open-label, single-arm, proof-of-concept study explored the feasibility of implementing extended-release naltrexone (380 mg monthly via intramuscular injection) plus medical management (physician-led counseling focusing on medication adherence and alcohol abstinence) into primary care settings for the treatment of alcohol dependence. A total of 72 subjects were enrolled in the study; 65 received at least one injection, and 40 received all three planned injections. By intent-to-treat analysis, median drinks per day decreased from 5.4 (95% CI, 4–7) to 3.4 (95% CI, 2–5). Among the 40 subjects receiving all three injections, median drinks per day decreased from 4.1 (95% CI, 3–6) to 0.5 (95% CI, 0–1.7).

#### Is acamprosate effective for alcohol dependence?

Rösner S and colleagues [55].

Prior studies have suggested that acamprosate may have some efficacy for maintaining abstinence in alcohol-dependent patients; however, certain large randomized trials have called its efficacy into question [56-59]. In this systematic review, the authors reviewed 24 randomized clinical trials representing 6915 subjects with alcohol dependence [55]. Treatment duration ranged from eight weeks to one year; all studies coupled medication with psychosocial treatments, and most studies required a period of abstinence prior to initiating study medication. Acamprosate increased self-reported abstinence in 25% of patients compared with 17% in the placebo group, with a relative risk of relapse to drinking of 0.86 (95% CI, 0.81-0.91); it also increased cumulative days abstinent by 11 days (95% CI, 5.1-16.8) but had no effect on heavy drinking. Three studies compared acamprosate with naltrexone and found no difference between the two medications with respect to outcomes.

#### Are there non-FDA approved agents that are effective for the treatment of alcohol dependence?

Paparrigopoulos T and colleagues [60].

Prior research suggests topiramate has efficacy for the treatment of alcohol dependence [61-63]; however, those studies focused on higher doses (150–300 mg daily), which is also associated with a greater potential for side effects. This open-label study [60] investigated the rate of relapse to alcohol use among alcohol-dependent patients randomized to low-dose topiramate (≤75 mg daily) (n = 30) or placebo (n = 60) after a period of inpatient detoxification. After 16 weeks, relapse to alcohol use was lower in the topiramate group (67%) compared with placebo (86%), (HR 0.57, p = 0.014).

Garbutt JC and colleagues [64].

A small, open-label study of the GABA agonist baclofen demonstrated reductions in drinks per drinking day and number of drinking days, as well as an increase in days abstinent, among people with alcohol dependence, despite a relatively high dropout rate due to side effects [65]. Additionally, baclofen has been evaluated as a potential treatment option for alcohol-dependent patients with cirrhosis given its lack of hepatotoxicity [66]. The authors of this study investigated the efficacy of baclofen compared with placebo for the treatment of alcohol dependence in patients with or without cirrhosis (N = 80) [64]. Seventy-six percent of patients completed the 12-week study. The authors found no differences in percentage of heavy drinking days (roughly 26% in both groups), time to first drink, time to relapse to heavy drinking, or craving between the baclofen and placebo groups.

#### Implications for practice

Opioid antagonists have efficacy for the treatment of alcohol dependence. Oral naltrexone is the most widely studied agent in this medication class and reduces relapse to alcohol use, relapse to heavy drinking, and heavy drinking days. Extended release injectable naltrexone is feasible to implement in primary care settings; however, larger controlled trials investigating drinking outcomes over time are warranted. Future investigations of opioid antagonists for the treatment of alcohol dependence should focus on comparisons with other medications and treatment modalities and in various clinical settings.

Despite only moderate efficacy, acamprosate, in conjunction with psychosocial treatments, should be considered a treatment option for alcohol dependence given the limited pharmacotherapeutic options and seriousness of the disease process. Both low-dose topiramate and baclofen may have a role in the treatment of alcohol dependence based on these and prior studies. However, larger randomized controlled trials are needed to adequately assess their efficacy.

## Competing interests

The authors declare that they have no competing interests for the work presented in this manuscript.

## Authors’ contributions

DAR authored the section on opioid pharmacotherapy, HVK the section on medical comorbidities, JMT the section on alcohol pharmacotherapy, AYW the section on prescription drug abuse, and AJG the section on screening and brief intervention. AJG coordinated the process of gathering and reviewing papers, DAR coordinated the writing of the manuscript. All authors read and approved the final manuscript.
